# Supplementary material for: Empirical methods for controlling false positives and estimating confidence in ChIP-Seq peaks
Source: BMC Bioinformatics. 2008 Dec 5;9:523. doi: 10.1186/1471-2105-9-523 (PMC2628906; doi:10.1186/1471-2105-9-523)
Supplement: Additional File 1 — A variety of html documents from the USeq web site detailing the available applications, their best usage, output file type descriptions, command line menus, etc. [file 1471-2105-9-523-S1.zip › USeqUserGuides/JavaDoc.zip!/JavaDoc/util/amazon/S3Manager.html]

S3Manager


|  |  |  |
| --- | --- | --- |
| **Overview** \| **Package** \| **Class** \| **Use** \| **Tree** \| **Deprecated** \| **Index** \| **Help** | |  |
| **PREV CLASS**   NEXT CLASS | **FRAMES**    **NO FRAMES**     **All Classes** |  |
| SUMMARY: NESTED \| FIELD \| CONSTR \| METHOD | DETAIL: FIELD \| CONSTR \| METHOD |  |


---


## util.amazon Class S3Manager

```
java.lang.Object
  util.amazon.S3Manager
```

---

``` public class S3Manager extends java.lang.Object ```

Class to handle Amazon S3 file upload and download using the JetS3t API.
See http://jets3t.s3.amazonaws.com/index.html

**Author:**
:   Nix

---

| **Field Summary** | |
| --- | --- |
| `org.jets3t.service.security.AWSCredentials` | `awsCredentials` |
| `org.jets3t.service.model.S3Bucket` | `bucket` |
| `java.lang.String` | `bucketName` |
| `boolean` | `connected` |
| `java.io.File` | `credentialsFile` |
| `org.jets3t.service.S3Service` | `s3Service` |
| `org.jets3t.service.multithread.S3ServiceSimpleMulti` | `simpleMulti` |
| `java.util.Map` | `threadedResults` |


**Constructor Summary**

`S3Manager(java.lang.String password,
java.lang.String bucketName,
java.io.File credentialsFile)`
  
          Constructor, after instantiating check that boolean.connected is true;


| **Method Summary** | |
| --- | --- |
| `boolean` | `connectAndTest()`             Instantiates connection objects and attempt to make a bucket if it doesn't exist to hold objects. |
| `boolean` | `delete(java.lang.String key)`             Attempts to delete keyed object from S3. |
| `boolean` | `delete(java.lang.String[] keys)`             Attempts to delete keyed objects from S3. |
| `boolean` | `download(java.io.File file)`             Uses the file name as the S3 key to fetch and save the file. |
| `boolean` | `download(java.io.File[] files)`             Uses the file names as the S3 keys to fetch and save the files. |
| `boolean` | `download(java.io.File dir, java.lang.String[] keys, java.lang.String[] fileNames)`             Downloads the keyed objects into the directory as files. |
| `boolean` | `download(java.lang.String uniqueProcessName, java.io.File dir, java.lang.String[] keys, java.lang.String[] fileNames)`             Downloads files from S3 using a different thread. |
| `java.lang.String[]` | `fetchAllS3ObjectKeyNames()`             Fetches the key names of the S3 objects. |
| `java.lang.String[]` | `fetchTimedURLs(int minutes, java.lang.String[] keys)`             Returns URLs to download particular objects from S3 that expire after a given amount of time. |
| `org.jets3t.service.security.AWSCredentials` | `getAwsCredentials()` |
| `org.jets3t.service.model.S3Bucket` | `getBucket()` |
| `java.lang.String` | `getBucketName()` |
| `java.io.File` | `getCredentialsFile()` |
| `org.jets3t.service.S3Service` | `getS3Service()` |
| `org.jets3t.service.multithread.S3ServiceSimpleMulti` | `getSimpleMulti()` |
| `java.util.Map` | `getThreadedResults()` |
| `boolean` | `isConnected()` |
| `boolean` | `loadCredentials()`             Attempts to create a AWSCredentials object from an encripted file. |
| `static void` | `main(java.lang.String[] args)` |
| `void` | `setAwsCredentials(org.jets3t.service.security.AWSCredentials awsCredentials)` |
| `void` | `setBucket(org.jets3t.service.model.S3Bucket bucket)` |
| `void` | `setBucketName(java.lang.String bucketName)` |
| `void` | `setConnected(boolean connected)` |
| `void` | `setCredentialsFile(java.io.File credentialsFile)` |
| `void` | `setS3Service(org.jets3t.service.S3Service service)` |
| `void` | `setSimpleMulti(org.jets3t.service.multithread.S3ServiceSimpleMulti simpleMulti)` |
| `void` | `setThreadedResults(java.util.Map threadedResults)` |
| `boolean` | `upload(java.io.File file)`             See upload (File[] files). |
| `boolean` | `upload(java.io.File[] files)`             Upload files to S3. |
| `boolean` | `upload(java.lang.String uniqueProcessName, java.io.File file)`             See threaded upload (String uniqueProcessName, File[] files). |
| `boolean` | `upload(java.lang.String uniqueProcessName, java.io.File[] files)`             Uploads files to S3 using a different thread. |
| `static boolean` | `writeEncryptedCredentialsFile(java.io.File credFile, java.lang.String password, java.lang.String accessKey, java.lang.String secretKey)`             Write a password protected encripted credentials file. |

**Methods inherited from class java.lang.Object**

`equals, getClass, hashCode, notify, notifyAll, toString, wait, wait, wait`

**Field Detail**

### bucketName

```
public java.lang.String bucketName
```

---


### credentialsFile

```
public java.io.File credentialsFile
```

---


### awsCredentials

```
public org.jets3t.service.security.AWSCredentials awsCredentials
```

---


### s3Service

```
public org.jets3t.service.S3Service s3Service
```

---


### simpleMulti

```
public org.jets3t.service.multithread.S3ServiceSimpleMulti simpleMulti
```

---


### bucket

```
public org.jets3t.service.model.S3Bucket bucket
```

---


### connected

```
public boolean connected
```

---


### threadedResults

```
public java.util.Map threadedResults
```


**Constructor Detail**

### S3Manager

```
public S3Manager(java.lang.String password,
                 java.lang.String bucketName,
                 java.io.File credentialsFile)
```

:   Constructor, after instantiating check that boolean.connected is true;

    **Parameters:**: `password` - - The password used to unlock the encrypted credentials file: `bucketName` - - Will attempt to create a bucket using this name if it doesn't exist, remember they must be unique, a good naming convention is the accessKey+".bucketName".: `credentialsFile` - - encrypted accessKey and secretKey, see writeEncryptedCredentialsFile()


**Method Detail**

### fetchAllS3ObjectKeyNames

```
public java.lang.String[] fetchAllS3ObjectKeyNames()
```

:   Fetches the key names of the S3 objects.
    Returns null if a problem is encountered.

---


### fetchTimedURLs

```
public java.lang.String[] fetchTimedURLs(int minutes,
                                         java.lang.String[] keys)
```

:   Returns URLs to download particular objects from S3 that expire after a given amount of time.

---


### upload

```
public boolean upload(java.io.File[] files)
```

:   Upload files to S3. This will overwrite any file in S3 with the same name.

---


### upload

```
public boolean upload(java.lang.String uniqueProcessName,
                      java.io.File[] files)
```

:   Uploads files to S3 using a different thread.
    Check status by reading the Value in threadedResults Map
    for the uniqueProcessName Key.
    If it says "Running" it's running, "Complete" it's complete,
    anything else is an error.

---


### upload

```
public boolean upload(java.lang.String uniqueProcessName,
                      java.io.File file)
```

:   See threaded upload (String uniqueProcessName, File[] files).

---


### upload

```
public boolean upload(java.io.File file)
```

:   See upload (File[] files).

---


### download

```
public boolean download(java.io.File dir,
                        java.lang.String[] keys,
                        java.lang.String[] fileNames)
```

:   Downloads the keyed objects into the directory as files.
    Usefull for renaming the keyed objects when saving as files.

    :   **Parameters:**: `dir` - - directory in which to save the files: `keys` - - String[] of S3 object key names: `fileNames` - - names to use in saving the files.

---


### download

```
public boolean download(java.lang.String uniqueProcessName,
                        java.io.File dir,
                        java.lang.String[] keys,
                        java.lang.String[] fileNames)
```

:   Downloads files from S3 using a different thread.
    Check status by reading the Value in threadedResults Map
    for the uniqueProcessName Key.
    If it says "Running" it's running, "Complete" it's complete,
    anything else is an error.

    :   **Parameters:**: `dir` - - directory to save files: `keys` - - object key names: `fileNames` - - what to rename each keyed object when saving as a file.

---


### download

```
public boolean download(java.io.File file)
```

:   Uses the file name as the S3 key to fetch and save the file.

---


### download

```
public boolean download(java.io.File[] files)
```

:   Uses the file names as the S3 keys to fetch and save the files.

---


### delete

```
public boolean delete(java.lang.String[] keys)
```

:   Attempts to delete keyed objects from S3.

---


### delete

```
public boolean delete(java.lang.String key)
```

:   Attempts to delete keyed object from S3.

---


### writeEncryptedCredentialsFile

```
public static boolean writeEncryptedCredentialsFile(java.io.File credFile,
                                                    java.lang.String password,
                                                    java.lang.String accessKey,
                                                    java.lang.String secretKey)
```

:   Write a password protected encripted credentials file.

---


### loadCredentials

```
public boolean loadCredentials()
```

:   Attempts to create a AWSCredentials object from an encripted file.

---


### connectAndTest

```
public boolean connectAndTest()
```

:   Instantiates connection objects and attempt to make a bucket if it doesn't exist to hold objects.

---


### main

```
public static void main(java.lang.String[] args)
```

---


### isConnected

```
public boolean isConnected()
```

---


### getAwsCredentials

```
public org.jets3t.service.security.AWSCredentials getAwsCredentials()
```

---


### setAwsCredentials

```
public void setAwsCredentials(org.jets3t.service.security.AWSCredentials awsCredentials)
```

---


### getBucket

```
public org.jets3t.service.model.S3Bucket getBucket()
```

---


### setBucket

```
public void setBucket(org.jets3t.service.model.S3Bucket bucket)
```

---


### getBucketName

```
public java.lang.String getBucketName()
```

---


### setBucketName

```
public void setBucketName(java.lang.String bucketName)
```

---


### getCredentialsFile

```
public java.io.File getCredentialsFile()
```

---


### setCredentialsFile

```
public void setCredentialsFile(java.io.File credentialsFile)
```

---


### getS3Service

```
public org.jets3t.service.S3Service getS3Service()
```

---


### setS3Service

```
public void setS3Service(org.jets3t.service.S3Service service)
```

---


### getSimpleMulti

```
public org.jets3t.service.multithread.S3ServiceSimpleMulti getSimpleMulti()
```

---


### setSimpleMulti

```
public void setSimpleMulti(org.jets3t.service.multithread.S3ServiceSimpleMulti simpleMulti)
```

---


### setConnected

```
public void setConnected(boolean connected)
```

---


### getThreadedResults

```
public java.util.Map getThreadedResults()
```

---


### setThreadedResults

```
public void setThreadedResults(java.util.Map threadedResults)
```


---


|  |  |  |
| --- | --- | --- |
| **Overview** \| **Package** \| **Class** \| **Use** \| **Tree** \| **Deprecated** \| **Index** \| **Help** | |  |
| **PREV CLASS**   NEXT CLASS | **FRAMES**    **NO FRAMES**     **All Classes** |  |
| SUMMARY: NESTED \| FIELD \| CONSTR \| METHOD | DETAIL: FIELD \| CONSTR \| METHOD |  |


---
